# A fully reconfigurable waveguide Bragg grating for programmable photonic signal processing

**DOI:** 10.1038/s41467-018-03738-3

**Published:** 2018-04-11

**Authors:** Weifeng Zhang, Jianping Yao

**Affiliations:** Microwave Photonic Research Laboratory, School of Electrical Engineering and Computer Science, University of Ottawa, 25 Templeton Street, Ottawa, ON K1N 6N5 Canada

## Abstract

Since the discovery of the Bragg’s law in 1913, Bragg gratings have become important optical devices and have been extensively used in various systems. In particular, the successful inscription of a Bragg grating in a fiber core has significantly boosted its engineering applications. However, a conventional grating device is usually designed for a particular use, which limits general-purpose applications since its index modulation profile is fixed after fabrication. In this article, we propose to implement a fully reconfigurable grating, which is fast and electrically reconfigurable by field programming. The concept is verified by fabricating an integrated grating on a silicon-on-insulator platform, which is employed as a programmable signal processor to perform multiple signal processing functions including temporal differentiation, microwave time delay, and frequency identification. The availability of ultrafast and reconfigurable gratings opens new avenues for programmable optical signal processing at the speed of light.

## Abstract (web summary)

Bragg gratings are versatile elements used to perform spectral filtering in optical circuits. Here, the authors develop a scalable, reconfigurable grating device which can be electrically tuned to actively change its behaviour and control of optical pathways, for use in photonic signal processing.

## Introduction

A fiber or waveguide Bragg grating is a one-dimensional optical device produced by periodic variation of the refractive index in the fiber core or the waveguide, which is able to reflect a particular wavelength of light and transmit all others^1^. By specifying the index modulation profile, the spectral response of a Bragg grating is determined^2^. Thanks to the simple configuration and unique filtering capability, a Bragg grating, as a versatile optical filter, which has enjoyed widespread applications in various scientific and industrial fields^3–8^. In particular, the discovery of fiber Bragg gratings (FBGs) by Hill and co-workers in 1978 has opened up an unprecedented opportunity for FBGs to perform optical signal processing which has revolutionized the fields of telecommunications and optical fiber sensing^9–12^. Benefiting from the rapid development of semiconductor technologies, significant advancement in silicon-based photonic integrated circuit (PIC) technologies has taken place since the beginning of this century^13^. A Bragg grating implemented on a silicon-based integrated photonic platform has been demonstrated^14–16^, and by integrating with other photonic devices on a same chip, an on-chip grating could achieve more advanced functionalities^17–20^. The spectral response of a Bragg grating, however, is predetermined by its index modulation profile, which is fixed. To date, most fiber-based or waveguide-based gratings are designed with a specific index modulation profile for a user-defined application. Although different mechanisms have been proposed to realize spectral tuning^21–24^, these tuning approaches are mainly limited to shifts of the center wavelength. For many applications, other spectral characteristics, such as spectral shape and phase response, should be tunable. For example, in microwave photonic signal processing^25–30^, grating devices are widely used to perform functions such as temporal differentiation^31–33^, filtering^34–36^, and true time delay^37–39^. To perform temporal differentiation and narrowband filtering, a phase-shifted Bragg grating is usually used; while to achieve true time delay with a broad operation bandwidth, a chirped grating is employed. For a programmable microwave signal processor, it is highly expected that a fully reconfigurable grating could be used to perform multiple functions. Recently, with the exponential growth of data traffic due to the multimedia services, the elastic optical network (EON) architecture is considered a promising solution for next-generation optical networking^40, 41^. Distinct from the fixed spectrum grid in the current optical networks, the spectrum grid in an EON is flexible. To address the need for flexible division of optical spectrum, a reconfigurable optical add-drop multiplexer (ROADM) is an essential component, which can generate elastic optical paths by reconfiguring its filter response^42^. A fast and fully reconfigurable grating is a strong candidate to fulfill this role. By field programming, the index modulation profile of the grating can be software defined, to reconfigure its spectral response for elastic channel requirements.

In this article, we propose an ultrafast and fully reconfigurable waveguide Bragg grating that is implemented on a silicon-on-insulator (SOI) platform. The key advantage of the grating is that it can be reconfigured electrically, and hence its spectral characteristics could be flexibly and precisely tailored for task-oriented applications. A proof-of-concept demonstration is made in which a grating is electrically reconfigured to be a phase-shifted, a uniform, and a chirped grating by field programming. By incorporating the grating in microwave photonic signal processing, a programmable signal processor to perform multiple processing functions including temporal differentiation, true time delay, and microwave frequency identification is experimentally demonstrated. The availability of such ultrafast and reconfigurable gratings opens new avenues for programmable optical signal processing at ultra-fast speed.

## Results

### Reconfigurable grating design

**Fig. 1:**
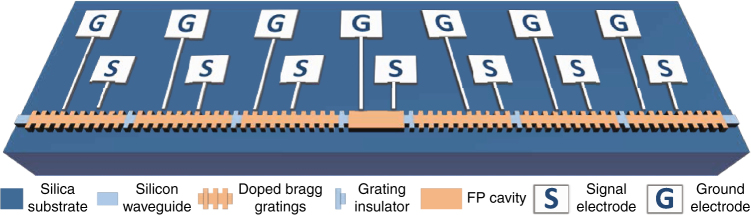
Schematic view of reconfigurable grating. The grating consists of multiple series-connected uniform Bragg grating sections and an FP cavity section in the center of the grating. Each uniform Bragg grating section incorporates an independent lateral PN junction, and between two neighboring sections there is an un-doped grating to function as an insulator. A pair of electrodes (Signal and Ground) are connected to each independent PN junction

Figure 1 illustrates the proposed reconfigurable grating. The grating consists of multiple series-connected uniform Bragg grating sections and a Fabry-Perot (FP) cavity section in the center of the grating. Each uniform Bragg grating section incorporates an independent lateral PN junction, and between two neighboring sections there is an un-doped grating to function as an insulator. Distributed electrodes are connected to the independent PN junctions. By applying a bias voltage to a PN junction, the refractive index of the grating in that particular section could be tuned locally based on free-carrier plasma dispersion effect^43^. Thus, the entire index modulation profile of the grating could be electrically reconfigured by field programming all the bias voltages, which enables the grating to have diverse spectral characteristics for diverse applications.

**Fig. 2:**
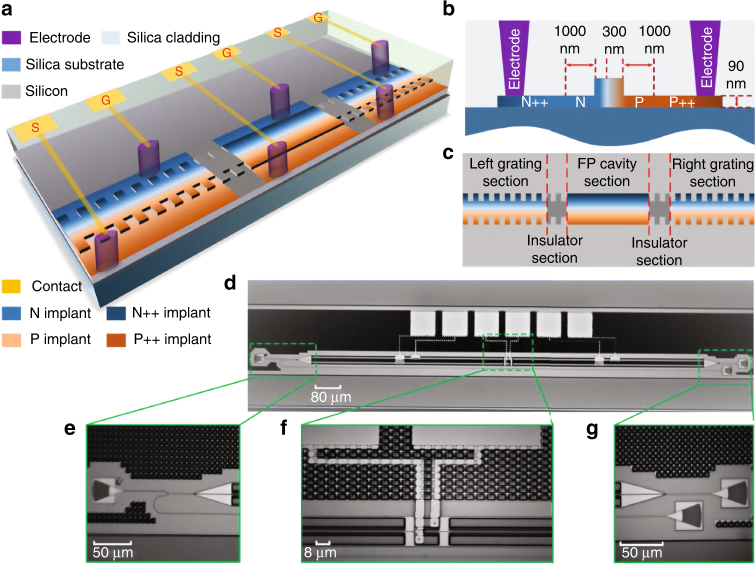
The designed reconfigurable grating. **a** Perspective view of the grating on a silicon chip. **b** Cross-sectional view of the grating rib waveguide. **c** Top-view of the grating. **d**-**g** Microscope camera images of the fabricated grating, the input grating coupler and compact Y-branch, the FP cavity section, and the transmission and reflection grating couplers

A proof-of-concept demonstration is made in which a reconfigurable grating is designed, fabricated and characterized. This grating has a symmetrical configuration, which consists of two identical uniform sub-grating sections (left and right) and a FP cavity section in the middle. Figure 2a illustrates the perspective view of the proposed grating on a silicon chip. Each section incorporates an independent lateral PN junction, which is connected to an individual pair of contacts for local refractive index tuning, and between two sections there is an un-doped grating acting as an insulator, to electrically isolate the two neighboring sections. Figure 2b, c shows the cross-sectional view of the grating waveguide and the top-view of the grating, respectively (see Methods section for more details about the grating design and layout).

The reconfigurable grating is fabricated at IME in a complementary metal-oxide-semiconductor (CMOS)-compatible process using 248-nm deep ultraviolet lithography. Figure 2d is a photograph of the fabricated grating captured by a microscope camera. Six contact windows are opened on the silica pads for three independent PN junctions. The entire device has a length of 1.560 mm and a width of 0.196 mm, giving a small footprint of 0.3 mm^2^. Figure 2e gives a zoom-in view of the input grating coupler and the compact Y-branch. Figure 2f gives a zoom-in view of the FP cavity. To make the design of the cavity section compliant with the stringent design rules, 20 extra grating periods are added to the two sides of the cavity in the FP cavity section. Between neighboring sections, 20 extra grating periods are also added to act as an insulator. Since the number of added grating periods is quite small, the grating effect could be ignored during the tuning. Figure 2g gives a zoom-in view of transmission and reflection grating couplers.

Each independent PN junction is individually tested by applying a bias voltage and measuring the reflection spectra of the grating. The measurement results (see Supplementary Note 1) show that by applying and tuning a bias voltage to each PN junction in each section, the local refractive index change in that particular section leads to an independent tuning of the spectral response of the grating. Thus, by field programming the three bias voltages applied to the three PN junctions, the index modulation profile of the grating can be reconfigured, and the grating spectral characteristics could be tailored in a precise and ultra-fast manner in a scale of nano-seconds.

### Reconfigured to be a phase-shifted grating

**Fig. 3:**
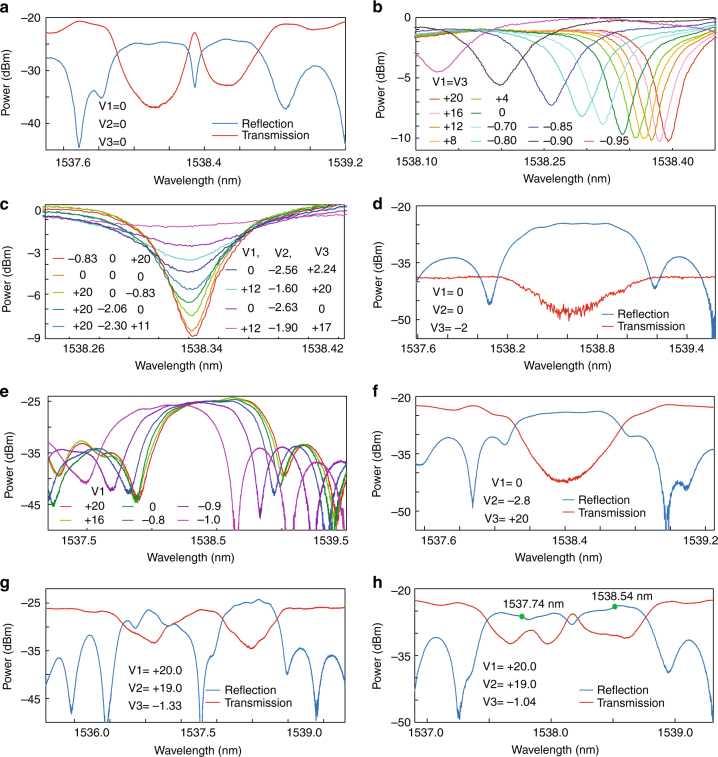
Measured reflection and transmission spectra. **a** Reflection and transmission spectra of the fabricated grating in the static state. **b** Notch wavelength shift when the bias voltages applied to the left and right sub-gratings vary synchronously. **c** Extinction ratio tuning while the notch wavelength is kept unchanged. **d** Reflection and transmission spectra when the grating is reconfigured to be a uniform grating. **e** Wavelength tuning of the uniform grating. **f** Reflection and transmission spectra when the device is reconfigured to be a uniform grating by increasing the cavity loss. **g** Reflection and transmission spectra when the device is reconfigured to be two independent uniform sub-gratings. **h** Reflection and transmission spectra when the device is reconfigured to be a chirped grating (The bias voltages and the power consumptions for different grating operation regimes are summarized in Supplementary Note 2.)

A phase-shifted waveguide Bragg grating can be implemented by introducing a phase shift in the center of a uniform grating. For the fabricated grating, the phase shift can be introduced by the FP cavity. Figure 3a shows the measured reflection and transmission spectra of the fabricated grating in the static state. As can be seen, a resonant window is located within the stopband in the transmission spectra (in red), which is a distinct feature of a phase-shifted Bragg grating. The measured reflection spectra (in blue) has a notch with a 3-dB bandwidth of 49 pm and an extinction ratio of 8.7 dB in the reflection band. The insertion loss of the fabricated device at the transmission port is 20.6 dB, which includes the fiber-to-fiber I/O coupling loss, the grating-induced loss, and the loss due to the ion implantations, while the insertion loss at the reflection port is 25.1 dB. Most of the insertion loss is caused by the grating couplers, which could be largely reduced by optimizing the design of the grating couplers.

Figure 3b shows a zoom-in view of the notch wavelength shift in the reflection band when two bias voltages applied to the PN junctions in the left and right sub-grating sections vary from +20 to −1 V synchronously. Thanks to the free-carrier plasma dispersion effect, the free-carrier concentration in the waveguide introduces a change in the reflective index of the waveguide. As the bias voltages vary, the grating spectral response is shifted. At a maximum reverse bias voltage of +20 V in the measurement, a notch wavelength shift as large as 54 pm is achieved. The power consumption is measured to be 2.31 μW. The wavelength shift rate is estimated to be 23.4 pm/μW. At a maximum forward bias voltage of −1.0 V in the measurement, the notch wavelength has a shift of 431 pm with a power consumption of 6.0 mW. During the shift, the extinction ratio of the notch becomes smaller as the bias voltages decrease. This is because the free-carrier-induced optical absorption loss is increased.

Figure 3c shows the tuning of the extinction ratio while the notch wavelength is maintained unchanged for different bias voltage combinations. It is known that in a conventional phase-shifted Bragg grating, it is not possible to tune the notch extinction ratio while maintaining the notch wavelength unchanged. In the fabricated grating, by field programming the three bias voltages, the notch wavelength shifts induced by the PN junctions could counteract. Thus, the notch wavelength can be kept unchanged, while different bias voltage combinations could lead to a different roundtrip loss, which would lead to a different notch extinction ratio. In Fig. 3c, an extinction ratio is 8.9 dB (maximum value) when the bias voltage combination is “−0.825, 0, +20 V” and a total power consumption is 260.8 μW, and an extinction ratio is 0.8 dB (minimum value) when the bias voltage combination is “+12, −1.9, +17 V” and a total power consumption is 12.48 mW. The notch extinction ratio tuning with the notch wavelength unchanged is a unique and advantageous feature of the reconfigurable grating when reconfigured to be a phase-shifted Bragg grating. The tuning range could be enhanced by increasing the section number or by employing an asymmetrical configuration.

### Reconfigured to be a uniform grating

The fabricated grating can be reconfigured as a uniform grating, which is realized by failing the optical confinement capability of the FP cavity, by applying a large forward bias voltage to the right PN junction. Figure 3d gives the measured reflection and transmission spectra of the grating when a large forward bias voltage of −2 V is applied to the right PN junction. The large forward bias voltage enables the injection of massive free-carriers into the waveguide, which would cause a heavy optical absorption loss and thus disable the reflection capability of the right sub-grating. As can be seen, there is one main peak in the reflection or a notch in the transmission spectra, which is a distinct feature of a uniform grating. Moreover, the insertion loss at the transmission port is much larger than the one at the reflection port, which is caused by the heavy optical absorption loss in the right sub-grating waveguide. In the reflection spectra, the reflection peak has a 3-dB bandwidth of 710 pm and a sidelobe suppression ratio of 9.05 dB at a power consumption of 5.59 mW. In addition, by tuning the bias voltage to the PN junction in the left sub-grating section, the center wavelength of the uniform grating could be tuned. As shown in Fig. 3e, when the bias voltage on the left PN junction varies, the grating spectral response is shifted. Specifically, at a maximum reverse bias voltage of +20 V, the spectral response is red shifted by 35 pm; at a maximum forward bias voltage of −1.0 V, the spectral response is blue shifted by 380 pm.

There is another approach to reconfigure the fabricated grating to be a uniform grating, which is realized by applying a large forward bias voltage to the cavity PN junction. Figure 3f gives the measured reflection and transmission spectra of the uniform grating when a forward bias voltage of −2.8 V is applied to the cavity PN junction. A large forward bias voltage enables the injection of massive free-carriers into the cavity to cause a heavy optical absorption loss. Thus, the optical confinement capability of the cavity would be severely undermined, in which the notch extinction ratio is heavily decreased and the notch wavelength is largely shifted to 1538.75 nm. As can be seen in Fig. 3f, in the reflection spectra, the 3-dB bandwidth of the main peak is 580 pm at a power consumption of 55 mW. The bandwidth becomes smaller than that of the uniform grating implemented by electrically disabling the right sub-grating. This is because the left and the right sub-gratings are working jointly. The step on either side of the peak is caused by the shifted notch of the weakened cavity and the shifted spectral response of the right sub-grating. The joint operation of the two sub-gratings has a strong reflectivity, which enables a flat top of the reflection peak, and the existing of steps on either side is of benefit to a sharp edge slope of the reflection peak. By programming voltages applied to the PN junctions, the fabricated grating could present some uncommon optical characteristics which are difficult to achieve by using a conventional grating. This is a unique feature of the fabricated grating.

Since the PN junctions in the left and right sub-grating sections can be independently controlled, the uniform sub-gratings in the two sections could be tuned independently. Figure 3g gives the measured reflection and transmission spectra of two uniform sub-gratings when a reverse bias voltage of +20 V is applied to the left PN junction, and a forward bias voltage of −1.33 V is applied to the right PN junction. Thus, the left sub-grating is red shifted and the right sub-grating is blue shifted, which reconfigures the fabricated grating to be two nonidentical uniform sub-gratings. As can be seen, there are two separate main reflection peaks in the reflection spectra. There is a clear difference between the two peaks. The reason for the difference is that the big forward bias voltage would induce a large optical absorption loss, which degrades optical performance of the right sub-grating. In addition, the nonuniformity between the two sub-gratings would also contribute to this difference.

As demonstrated, the fabricated grating could be reconfigured to be a uniform grating by programming the bias voltages. The optical performance of the grating could be further improved if advanced fabrication technology is used since the currently available standard foundry fabrication process imposes a tough limitation on the resolution of the grating index modulation.

### Reconfigured to be a chirped grating

Since the uniform sub-gratings in the left and right sections could be independently tuned, by shifting the spectral response of one of the two uniform sub-gratings with different bias voltages, the device could be reconfigured to be a chirped grating. Figure 3h presents the measured reflection and transmission spectra of the chirped grating when a maximum reverse bias voltage of +20 V is applied to the left PN junction and a forward bias voltage of −1.04 V is applied to the right PN junction. As can be seen, the 3-dB bandwidth of the spectra is increased to be 1.29 nm, which is much larger than that of the uniform grating. The power consumption is measured to be 4.16 mW. Due to the existence of the FP cavity, there is still a shallow notch in the middle of the spectra. Since its extinction ratio is quite small, the notch impact could be neglected. By increasing the grating length and dividing the grating into more sections, the fabricated grating would have a better optical performance in terms of the group delay and chirp rate when reconfigured to be a chirped grating.

In summary, thanks to the strong reconfigurability enabled by the three independently controllable PN junctions, by applying different bias voltages, the fabricated grating could vary its index modulation profile to present diverse spectral characteristics. A phase-shifted, a uniform and a chirped grating has been demonstrated. Such a reconfigurable grating device overcomes the long-standing limitation of conventional grating devices that have fixed modulation index profiles and presents overwhelming advantages in terms of strong and ultra-fast reconfigurability, compact size, and low power consumption.

### Programmable microwave photonic signal processor

**Fig. 4:**
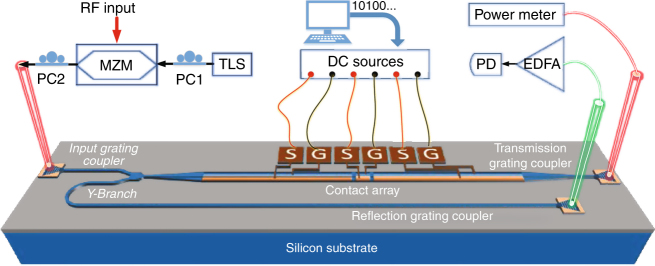
Schematic view of a programmable microwave signal processor. The experimental set-up consists of a tunable laser source (TLS), a polarization controller (PC), a Mach-Zehnder modulator (MZM), an erbium-doped fiber amplifier (EDFA), and a photodetector (PD)

By incorporating the reconfigurable grating in a typical microwave photonic system, a microwave signal processor could be realized. Thanks to the ultrafast full reconfigurability of the grating, this signal processor could be programmed to perform multiple functions. Figure 4 shows the experimental set-up. Three photonic signal processing functions, including tunable fractional-order temporal differentiation, microwave true time delay and microwave frequency identification, are experimentally demonstrated.

### Function of temporal differentiation

**Fig. 5:**
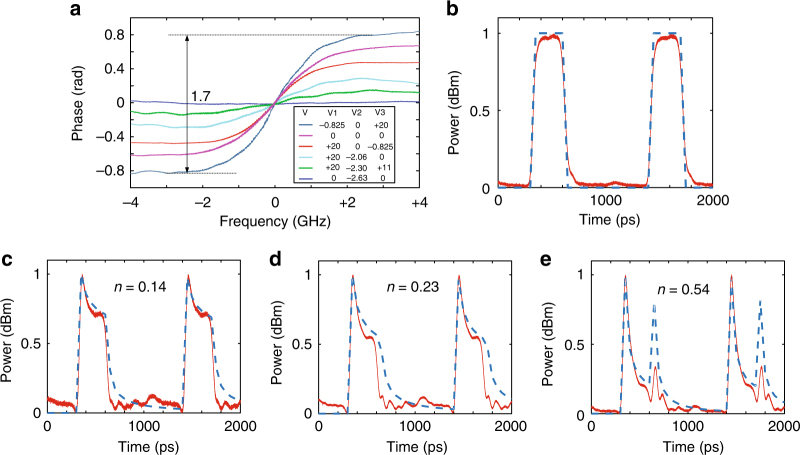
Experimental result of temporal differentiation. **a** Tunable phase jump of the reconfigurable grating. **b** Generated in red and simulated in blue rectangular pulse train. **c**–**e** The differentiated rectangular pulse with a fraction order of 0.14, 0.23, and 0.54

Thanks to the strong reconfigurability of the fabricated grating, the notch extinction ratio can be tuned while maintaining the center wavelength of the notch unchanged, thus it is advantageous to use the grating to realize a fractional-order tunable photonic temporal differentiator^44, 45^. Figure 5a shows the phase response of the grating when it is reconfigured as a phase-shifted Bragg grating. As can be seen, by programming the three bias voltages, the phase jump at the notch center could be tuned from 1.7 to 0, corresponding to a fractional order of 0.54 to 0. The phase jump tuning range could be increased by employing an asymmetrical configuration in the grating design. The temporal width of an individual microwave pulse is 290 ps, as shown in Fig. 5b in which a simulated rectangular pulse train (in dashed blue) is also given for comparison. Figure 5c–e shows three differentiated pulses corresponding to three differentiation orders of 0.14, 0.23, and 0.54. Simulations are performed to calculate the temporal differentiation of the input rectangular pulse with three differentiation orders of 0.14, 0.23, and 0.54. The experimental results agree well with the simulation results. The slight mismatch between the simulation and experimentally generated waveforms is due to the imperfect shape of the generated rectangular pulse compared with an ideal rectangular pulse. The key advantage of using the reconfigurable grating to implement tunable differentiation is that only the differentiation order is tuned and the center wavelength of the notch is fixed, a feature highly needed for signal processing in optical networks where the wavelengths are fixed.

### Function of microwave time delay

**Fig. 6:**
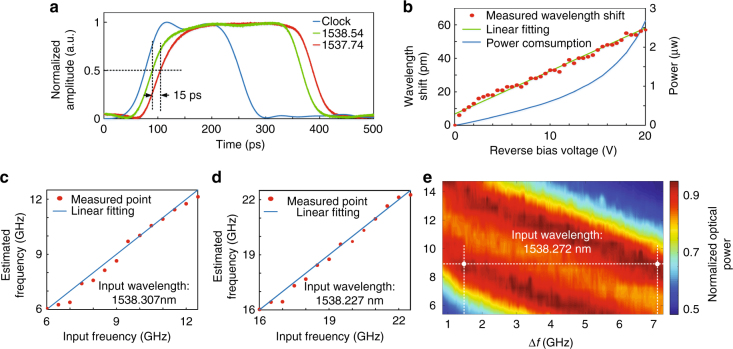
Experimental results of microwave time delay and frequency identification. **a** Time-domain measurement when the fabricated grating is reconfigured to be a chirped grating. **b** Peak wavelength shift and power consumption with the bias voltages. **c** Estimated frequencies when the wavelength of the optical carrier is 1538.307 nm. **d** Estimated frequencies when the wavelength of the optical carrier is 1538.227 nm. **e** Two-frequency measurement test when the wavelength of the optical carrier is swept from 1538.227 to 1538.307 nm

When the fabricated grating is reconfigured to be a chirped grating, it can be used as an optical true time delay line to generate tunable microwave time delays. To demonstrate the time delay function, a microwave rectangular pulse with a temporal width of 290 ps is modulated on two optical carriers with different wavelengths and sent to the grating. Two time-delayed optical pulses are reflected by the grating at different locations corresponding to two different time delays. After photodetection, the two time-delayed optical pulses are converted to two microwave pulses. Figure 6a shows the generated microwave pulse captured by using a sampling oscilloscope. The two pulses experience two different time delays, since they are carried by two different wavelengths at 1537.74 and 1538.54 nm. Specifically, the pulse carried by the optical wavelength at 1537.74 nm is reflected by the right sub-grating from its center. The pulse carried by the optical wavelength at 1538.54 nm is reflected by the left sub-grating from its center. The time delay difference is 15 ps, which is consistent with the theoretically calculated group delay response of the chirped grating. In order to have a large time delay, the length of the grating needs to be increased, and more independent grating sections could be incorporated for high-level tuning in terms of group delay and chirp rate.

### Function of frequency identification

Thanks to the ultra-large bandwidth offered by modern optics, microwave frequency measurement based on photonic techniques has attracted extensive research interest and numerous approaches have been reported^46^. The reconfigurable grating can also be used for wideband microwave frequency identification. Figure 6b presents the transmission peak wavelength shift with two reverse bias voltages at the left and right PN junctions increased synchronously when the grating is reconfigured to be a phase-shifted Bragg grating. The green line is obtained by linear fitting, which verifies the peak wavelength shift has a linear relationship with the reverse voltage. The blue line gives the power consumption at different bias voltages. Thanks to the linear wavelength shift with the bias voltage, by sweeping the two reverse bias voltages synchronously and recording the transmitted optical power, the optical frequency could be identified by reading the bias voltage corresponding to the frequency with the highest transmitted optical power.

A single-frequency measurement test is firstly performed. Figure 6c shows the estimated frequencies when the wavelength of the optical carrier is 1538.307 nm. A microwave signal with a frequency tunable from 6 to 12.5 GHz is generated and applied to the modulator. As shown in Fig. 6c, the measured frequencies match well with the actual frequencies. The errors between the measured values and actual frequencies are limited within ±0.60 GHz. By relocating the carrier wavelength further away from the transmission peak wavelength, it could be used to measure a microwave signal at a higher frequency. Figure 6d shows the estimated frequencies when the wavelength of the optical carrier is 1538.227 nm and a microwave signal with a frequency tunable from 16 to 22.5 GHz is applied. As can be seen, the measured frequencies match well with the actual frequencies again. The errors between the measured values and actual frequency values are limited within ±0.55 GHz.

To further evaluate the performance of the measurement system, a two-frequency measurement test is performed. Two microwave signals at 10.5 and 16.5 GHz are combined and applied to the modulator. Figure 6e shows the spectrogram of the transmitted optical power after voltage sweeping for different carrier wavelength. The *x*-axis is the frequency shift corresponding to each bias voltage, and the *y*-axis is the normalized frequency which is calculated by subtracting the carrier wavelength from the initial static wavelength. The real frequency is the summation of the normalized frequency and the frequency shift. As can be seen, when the carrier wavelength is located at 1538.272 nm, the two microwave frequencies is estimated to be 10.32 and 15.63 GHz, which match well with the actual frequencies. The two-frequency measurement test verifies that such a system could be used to identify two frequencies simultaneously. The measurement resolution is determined by the transmission selectivity or the Q-factor. To further enhance the performance of the system for multi-frequency measurement, the transmission selectivity of the grating needs to be significantly improved.

## Discussion

Thanks to the independently controllable PN junctions, the fabricated grating could be reconfigured to be a uniform grating, a phase-shifted grating, and a chirped grating by programming the bias voltages. If an asymmetrical configuration is employed and more independent grating sections are incorporated, the grating would provide more flexibility in terms of tuning and reconfigurability. In addition, with the use of advanced fabrication technology, the optical performance of the grating could be significantly improved.

A reconfigurable grating can find numerous applications. An application example is its use for programmable signal processing. Three signal processing functions including temporal differentiation, true time delay, and microwave frequency identification have been demonstrated. In fact, a programmable microwave signal processor based on a reconfigurable grating could perform other signal processing functions such as microwave filtering, temporal integration, and Hilbert transformation. Compared with the signal processor reported in ref. ^47^, our proposed programmable signal processor is simpler but with stronger reconfigurability. The reconfigurability of the proposed grating is similar to that of the 2D mesh networks reported in refs. ^48, 49^, where multiple optically interconnected cells that were thermally tunable were used to achieve reconfigurability, but the structures were more complicated. In addition, since the frequency response of our proposed grating is not periodic (not a finite impulse response filter), it is more desirable for system applications.

In addition to its use in microwave signal processing, the proposed grating could also be employed for arbitrary microwave waveform generation. For example, it can be used as a spectral shaper to generate a chirped microwave waveform for radar and other imaging applications^50, 51^. An array of such gratings can also be used as a beamforming network to generate true time delays for wideband squint-free beam steering^52^. By increasing the number of independent sub-grating sections, the functionalities of the signal processor could be further increased, and the performance could be enhanced.

In conclusion, we have proposed a grating that could be electrically reconfigurable by field programming at ultra-fast speed. A proof-of-concept demonstration was made in which a grating with two sub-grating sections and a FP cavity section was designed, fabricated, and characterized. The grating was electrically reconfigured to be a phase-shifted, a uniform, and a chirped grating by programming the bias voltages. An application for signal processing has been performed in which a signal processor to perform temporal differentiation, true time delay, and microwave frequency identification was experimentally demonstrated. The reconfigurable grating concept opens new avenues for on-chip gratings for multi-functional applications.

## Methods

### Grating design and layout

The grating is produced by creating periodic corrugations on the rib sidewall. To support a single fundamental TE mode operation, the rib waveguide is designed to have a width of 500 nm, a height of 220 nm, and a thickness of 90 nm. To have a higher tuning efficiency, an asymmetrical lateral PN junction is adopted. As shown in Fig. 2b, the PN junction is slightly shifted to the left from the center of the waveguide by 50 nm, to increase the mode overlap with the p-type doping region, since the free-carrier plasma dispersion effect is more sensitive to the change of the free-hole concentration. Additional p++ and n++ implantations, 1 μm away from the rib to minimize absorption losses, are utilized for ohmic contact formation. To enable the grating to operate in the optical communication window at C band, the grating period Λ is designed to be 310 nm. The duty cycle is selected to be 50%, and the periodic sidewall corrugations have a depth of 100 nm. The length of each grating section is 607.29 μm, and that of the FP cavity is 2.40 μm, which is allocated at the center of the grating. Three TE-mode grating couplers are used to couple light between the chip and the input and output fibers, and a compact Y-branch is used to collect the reflected light. To minimize the chip footprint and reduce the bending loss, a strip waveguide is used to guide the optical signal between the grating coupler and the gratings. Since the grating is implemented in a rib waveguide, a double-layer linear taper waveguide with a length of 50 μm is used for the mode transition between the strip and rib waveguides.

### Temperature-stabilized setup

In order to control and stabilize the chip temperature, a thermoelectric-cooler (TEC) was used on which the silicon chip was located. A thermistor was placed adjacent to the silicon chip, to measure and provide a feedback temperature to a commercial TEC controller, which was employed to control and stabilize the chip temperature at 23 °C during the experiment.

### Data availability

The data that support the findings of this study are available from the corresponding author upon request.

## Electronic supplementary material

Supplementary Information
