# Supplementary material for: A fully reconfigurable waveguide Bragg grating for programmable photonic signal processing
Source: Nat Commun. 2018 Apr 11;9:1396. doi: 10.1038/s41467-018-03738-3 (PMC5895633; doi:10.1038/s41467-018-03738-3)
Supplement: Supplementary file 1 — Supplementary Information [file 41467_2018_3738_MOESM1_ESM.pdf]

A fully reconfigurable waveguide Bragg grating for programmable photonic signal processing

Weifeng Zhang and Jianping Yao

Microwave Photonic Research Laboratory, School of Electrical Engineering and Computer Science, University of Ottawa, 25 Templeton Street, Ottawa, Ontario, Canada K1N 6N5

Supplementary Note 1: PN junction test

The tuning of each independent PN junction is tested by applying a bias voltage to the junction and measuring the spectra of the grating. To clearly illustrate the spectral shift during the test, a zoom-in view of the spectral response at the notch in the reflection band is presented.

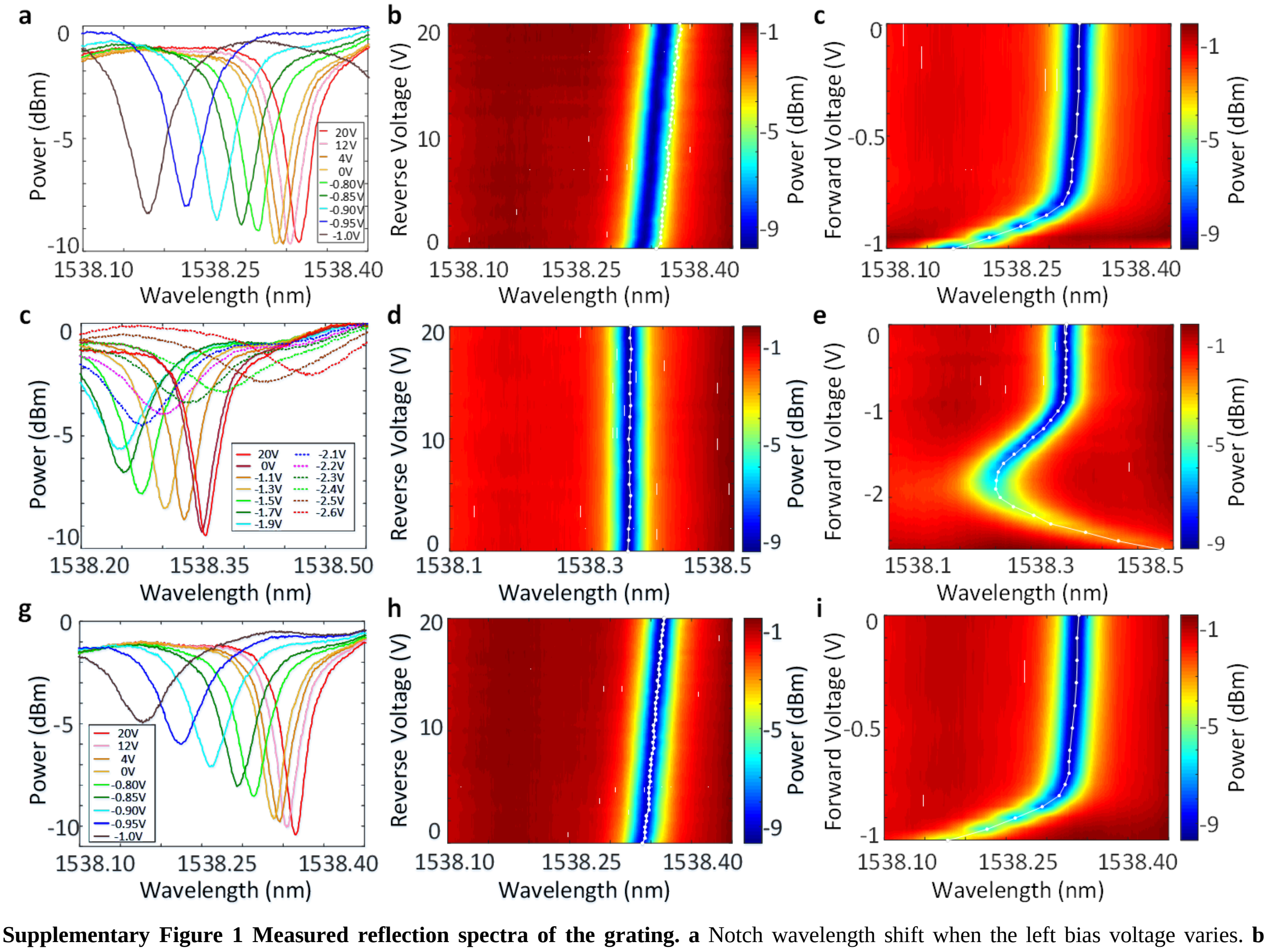

Supplementary Figure 1 Measured reflection spectra of the grating. a Notch wavelength shift when the left bias voltage varies. **b** Spectrogram of the notch center when the left PN junction is reverse biased. **c** Spectrogram of the notch center when the left PN junction is forward biased. **d** Notch wavelength shift when the FP cavity bias voltage varies. **e** Spectrogram of the notch center when the cavity PN junction is reverse biased. **f** Spectrogram of the notch when the cavity PN junction is forward biased. **g** Notch wavelength shift when the right bias voltage varies. **h** Spectrogram of the notch center when the right PN junction is reverse biased. (**i**) Spectrogram of the notch center when the right PN junction is forward biased.

Firstly, a bias voltage is applied to the PN junction in the left sub-grating section, while the PN junctions in the right sub-grating and the FP cavity sections are kept in the static state. Supplementary Figure 1 (a) shows the notch wavelength shift

when the bias voltage varies from +20 to -1 V. When the bias voltage applied to the left PN junction varies, the refractive index of the left sub-grating section is changed, which breaks the symmetry of the device configuration, and hence the notch of the grating is shifted. Supplementary Figure 1 (b) illustrates the spectrogram when the left PN junction is reverse biased, in which the white line depicts the notch center wavelength obtained by Lorentzian fitting. As can be seen, when reverse biased, the notch wavelength is red-shifted as the bias voltage is increasing. The wavelength shift presents a linear relationship with the bias voltage. The reason is that when the PN junction is reverse biased, the free-carrier concentration is very small due to the extraction. By using small signal approximation, the refractive index change of the waveguide has a linear relationship with the applied bias voltage. Therefore, the notch wavelength is linearly shifted with the reverse bias voltage. To prevent the breakdown of the PN junction, the maximum applied bias voltage is chosen to be +20 V in the measurement, which has a maximum notch wavelength shift of 29 pm with a power consumption of 1.65 μW. The wavelength shift rate is estimated to be 15.6 pm/μw. Supplementary Figure 1 (c) illustrates the spectrogram when the left PN junction is forward biased, and again the white line depicts the notch center wavelength obtained by Lorentzian fitting. As can be seen, when forward biased, the notch is blue-shifted as the bias voltage is increasing, and the relationship between the wavelength shift and the bias voltage is nonlinear. The bigger the voltage increases, the larger the notch wavelength is shifted. At the maximum forward bias voltage of -1.0 V in the measurement, the notch wavelength has a shift of 155 pm with a power consumption of 3.3 mW.

Secondly, a bias voltage is applied to the PN junction in the FP cavity section, while the PN junctions in the left and right sub-grating sections are kept in the static state. Supplementary Figure 1 (d) shows the notch wavelength shift when the bias voltage varies from +20 to -1 V. When the bias voltage applied to the cavity PN junction varies, the refractive index of the FP cavity section is tuned, which leads to a change in the cavity length and thus a shift in the notch wavelength of the grating. Supplementary Figure 1 (e) illustrates the spectrogram when the cavity PN junction is reverse biased, in which the white line depicts the notch center wavelength obtained by Lorentzian fitting. As can be seen, when reverse biased, the notch wavelength is red-shifted as the bias voltage is increasing, and the relationship between the notch wavelength shift and the reverse bias voltage keeps linear. However, since the cavity length is quite small, when reverse biased the free-carrier concentration change is very small, which results in a small notch wavelength shift. When the PN junction is biased at the maximum voltage of +20 V in the measurement, a maximum notch wavelength shift of 5 pm is achieved with a power consumption of 0.23 μW. The wavelength shift rate is estimated to be 22.2 pm/μw. Supplementary Figure 1 (f) illustrates the notch spectrogram when the cavity PN junction is forward biased, and again the white line depicts the notch center wavelength obtained by Lorentzian fitting. As can be seen, when forward biased, the notch is firstly blue-shifted as the bias voltage is increasing. When the bias voltage reaches a threshold value, the notch would start to red-shift. The reason is that when the PN junction is forward biased, the free-carrier plasma dispersion induced blue-shift is competing with the thermal-optic induced red-shift, since the PN junction is a variable resistor generating heat when a forward bias is applied. At the beginning, the free-carrier plasma dispersion effect dominates, which results in the blue-shift. However, as the voltage continues increasing, thermo-optic effect would dominate, which increases the effective refractive index and results in wavelength red-shift finally. At the threshold value of the forward bias voltage at -1.9 V in the measurement, the notch wavelength shift is 99 pm with a power consumption of 10.1 mW. Furthermore, with the forward bias voltage increasing, the free-carrier induced optical absorption loss in the cavity is increased, which heavily degrades optical performance of the cavity in terms of extinction ratio and optical confining-capability, illustrated clearly in the figure.

Finally, a bias voltage is applied to the PN junction in the right sub-grating section, while the PN junctions in the left sub-grating and FP cavity sections are kept in the static state. Supplementary Figure 1 (g) shows the notch wavelength shift when the bias voltage varies from +20 to -1 V. When the bias voltage applied to the right PN junction varies, the refractive index of the right grating section is tuned, which breaks the symmetry of the device configuration and hence the notch of the grating is shifted. Supplementary Figure 1 (h) illustrates the spectrogram when the right PN junction is reverse biased, in which the white line depicts the notch center wavelength obtained by Lorentzian fitting. As can be seen, under reverse bias, the notch is red-shifted as the bias voltage increases, and the wavelength shift is linear with the bias voltage. When the maximum bias voltage of +20 V is applied in the measurement, the notch wavelength shift has a maximum value of 27 pm with a power consumption of 1.554 μW. The wavelength shift rate is estimated to be 17.4 pm/μw. Supplementary Figure 1 (i) illustrates the spectrogram when the left PN junction is forward biased, and again the white line depicts the notch center wavelength obtained by Lorentzian fitting. As can be seen, under forward bias, the notch is blue-shifted as the bias voltage increases, and the relationship between the wavelength shift and the bias voltage is nonlinear. The bigger the voltage increases, the larger the notch wavelength is shifted. At the maximum forward bias voltage of -1.0 V in the measurement, the notch wavelength has a shift of 161 pm with a power consumption of 2.7 mW.

Since the fabricated reconfigurable grating has a symmetrical configuration, when the PN junction in the left or right sub-grating section is biased, almost the same grating spectral tuning is achieved in terms of notch wavelength shift and power consumption. However, there is an obvious difference in the extinction ratio variation of the notch between the two situations when the bias voltage is applied to the left and right PN junctions, as shown in Supplementary Figure 1 (a) and (g). It is

known that the notch in the reflection band occurs due to the destructive interference between the reflected optical signals from the left and right sub-gratings. Since the left and right sub-grating sections have the same grating structure, the reflected optical power from the left sub-grating section is always stronger than that from the right sub-grating section. This is because the reflected optical signal from the right sub-grating section travels a round trip along the entire grating. When the left PN junction is biased, the extinction ratio of the notch has a small degradation of 1.31 dB as the bias voltage varies from +20 to -1.0 V. The reason is that the change in the left sub-grating section would impose the same impact on the reflected optical signals from the left and right sub-gratings. On the contrary, when the right PN junction is biased, only the optical signal reflected from the right sub-gratings will suffer from the change in the right section. As shown in Supplementary Figure 1 (g), the extinction ratio of the notch is heavily deteriorated from 9.52 to 3.40 dB as the bias voltages varies. This is because the right PN junction when forward biased induces a large optical absorption loss, which heavily weakens the reflectivity of the right sub-grating. Thus, the optical power reflected from the left and right sub-gratings could not cancel and a weak destructive interference happens. In addition, grating nonuniformity between the left and right sub-grating sections imposed by fabrication imperfections could also contribute to the notch variation difference, which could be eliminated by using advanced fabrication technology. Furthermore, as the forward bias voltage continues increasing, the optical absorption loss would increase severely, which would heavily weaken the grating reflection effect or fail the grating ultimately.

From the measurements, the reconfigurable grating can be independently tuned by tuning the bias voltage to a PN junction. Thus, by field programming the bias voltages applied to the three PN junctions, the index modulation profile of the grating can be controlled, to tailor the spectral characteristics of the grating in an ultra-fast manner in a scale of nano-seconds.

Supplementary Note 2: Different bias voltages corresponding to different operation regimes of the reconfigurable grating

By field programming the bias voltages, the grating could be electrically reconfigured to be a phase-shifted, a uniform and a chirped grating. By incorporating this grating in a typical microwave photonic system, a programmable microwave signal processor can be implemented to perform multiple signal processing functions including temporal differentiation, microwave delay time and frequency identification. The bias voltages and the power consumptions are summarized in Supplementary Table 1 for the grating to operate in different regimes.

Supplementary Table 1 The bias voltages when the grating is reconfigured to be a phase-shifted, a uniform and a chirp grating

Operation regimes		Bias voltage on left sub-grating section V1 (V)	Bias voltage on FP section V2 (V)	Bias voltage on right sub-grating section V3 (V)	Power consumption
Phase-shifted grating	Fig. 3(a)	0	0	0	0 μW
	Fig. 3(b)	+20	0	+20	2.31 μW
		+16	0	+16	1.25 μW
		+12	0	+12	0.74 μW
		+8	0	+8	0.45 μW
		+4	0	+4	0.14 μW
		0	0	0	0 μW
		-0.70	0	-0.70	36.44 μW
		-0.80	0	-0.80	0.34 mW
		-0.85	0	-0.85	0.87 mW
		-0.90	0	-0.90	1.94 mW
		-0.95	0	-0.95	3.53 mW
	Fig. 3(c)	-0.83	0	+20	0.26 mW
		0	0	0	0 mW
		+20	0	-0.83	0.22 mW
		+20	-2.06	0	17.8 mW
		+20	-2.30	+11	21.4 mW
		0	-2.56	+2.24	42.3 mW
		+12	-1.60	+20	5.1 mW
		0	-2.63	0	44.7 mW

		+12	-1.90	+17	12.5 mW
Uniform grating	Fig. 3(d)	0	0	-2	5.6 mW
	Fig. 3(e)	+20	0	-2	5.6 mW
		+16	0	-2	5.6 mW
		0	0	-2	5.6 mW
		-0.80	0	-2	5.8 mW
		-0.90	0	-2	6.7 mW
		-1.00	0	-2	9.0 mW
	Fig. 3(f)	0	-2.8	+20	51.7 mW
	Fig. 3(g)	+20	+19	-1.33	27.5 mW
Chirped grating	Fig. 3(h)	+20	+19	-1.04	5.4 mW
